# A Probable Case of Mirogabalin-Induced Neutropenia

**DOI:** 10.7759/cureus.10182

**Published:** 2020-09-01

**Authors:** Saeko Takahashi, Akihiko Ogata, Morio Nakamura

**Affiliations:** 1 Department of Pulmonary Medicine, Tokyo Saiseikai Central Hospital, Tokyo, JPN

**Keywords:** mirogabalin, neutropenia, cancer, pain

## Abstract

Mirogabalin is a novel ligand for the α2δ subunit of voltage-gated calcium channels and is used to treat neuropathic pain in a similar manner to pregabalin. Although the frequency of pregabalin-induced neutropenia has been reported as 0.3%-1%, mirogabalin-induced neutropenia has not previously been reported in the literature. Herein, we report what we believe is the first case of neutropenia induced by mirogabalin. A 77-year-old woman with squamous cell carcinoma of the lung had been taking mirogabalin at 10 mg/day for six weeks prior to admission to our hospital. She had received two courses of chemotherapy with carboplatin and nanoparticle albumin-bound (nab)-paclitaxel for lung cancer until four months before admission, followed by two courses of nivolumab until one month before admission. The patient was hospitalized for urinary tract infection (UTI), which improved with oral amoxicillin/clavulanic acid at 500/125 mg three times daily for five days (until the fifth hospital day). After that, she underwent rehabilitation to improve muscle strength. During rehabilitation, neutropenia (1,278/µL) was noted, acetaminophen and mexiletine were ceased, and filgrastim was started on hospital day 17. The neutrophil count was 755/µL on hospital day 18. Mirogabalin was discontinued on hospital day 19. The neutrophil count fell to 320/µL and 118/µL on hospital day 20 and day 21, respectively, and recovered to 1,064/µL on hospital day 24. Acetaminophen and mexiletine were resumed on hospital day 31 and neutropenia has not recurred since.

## Introduction

Mirogabalin is a novel ligand for the α2δ subunit of voltage-gated calcium channels and is used to treat neuropathic pain in a manner similar to pregabalin. The reported incidence of pregabalin-induced neutropenia is 0.3%-1% [1], and the pathology is likely caused by direct damage to myeloid cells. Here, we report what we believe is the first case of mirogabalin-induced neutropenia.

## Case presentation

A 77-year-old woman with lung squamous cell carcinoma (cT4N2M1a, stage IVA), diagnosed six months earlier, was admitted to our hospital with urinary tract infection (UTI). She had received two courses of carboplatin (area under the curve: 5, day 1) and nab-paclitaxel (100 mg/m^2^, days 1 and 8) for lung cancer until four months before admission, followed by two courses of nivolumab at a fixed dose of 240 mg every two weeks until one month before admission. She had been taking acetaminophen at 3,000 mg/day for six months, hydromorphone at 6 mg/day for five months, and mexiletine at 150 mg/day and mirogabalin at 10 mg/day for six weeks until the day of hospitalization. UTI improved with oral amoxicillin/clavulanic acid at 500/125 mg three times daily for five days (until the fifth hospital day). After that, she underwent rehabilitation to improve muscle strength. Neutropenia (1,278/µL) was observed, and acetaminophen and mexiletine were discontinued on hospital day 17. Blood tests showed no abnormalities in other cell lineages and normal liver and renal function. Subcutaneous injection of filgrastim at 75 mg/day was started and administered for eight days (up to day 24). The neutrophil count was 755/µL on hospital day 18, and mirogabalin was discontinued on hospital day 19. The neutrophil count had fallen to 320/µL and 118/µL on hospital day 20 and day 21, respectively, and recovered to 1,064/µL on hospital day 24. Due to the onset of fever on hospital day 20, cefepime at 2 g q12h was started and continued for six days. Acetaminophen and mexiletine were resumed on hospital day 31, and neutropenia has not recurred since.

With respect to other pathologies that may cause neutropenia, the clinical course, as well as the absence of abnormalities in other cell lineages and absence of splenomegaly, suggests that the patient was unlikely to have had a hematologic malignancy or aplastic anemia. The patient had no complications of collagen diseases such as systemic lupus erythematosus. As the causative agent of the UTI was *Proteus mirabilis* and her food intake was normal, it was also considered unlikely that the UTI or nutritional disorder caused neutropenia.

**Figure 1:**
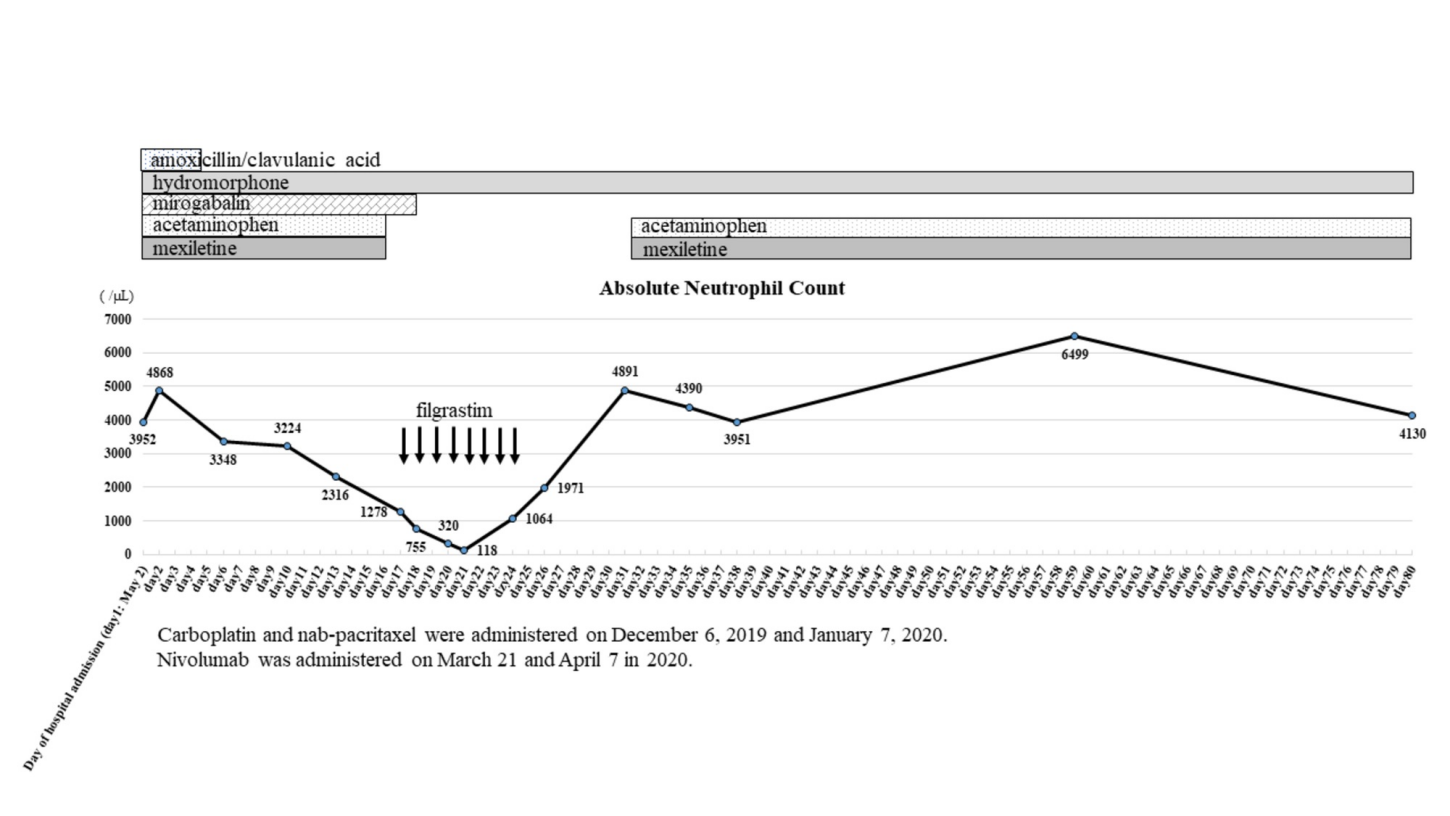
Changes of absolute neutrophil count after hospitalization

## Discussion

Although the patient had not undergone bone marrow examination and had been taking hydromorphone, acetaminophen, mexiletine, and mirogabalin, we attributed the neutropenia to mirogabalin after considering the clinical course. Like pregabalin, this recently developed agent is a ligand for the α2δ subunit of voltage-gated calcium channels. The incidence of pregabalin-induced neutropenia is reportedly 0.3%-1% [1]. Although the package insert for mirogabalin indicates one case of neutropenia [2], this adverse effect has not been published in a case report. In contrast, pregabalin-induced neutropenia starting two days after treatment initiation has been reported by Bozikas et al. [3], with the neutrophil count recovering three days after discontinuing pregabalin. The authors suggested that pregabalin-induced toxicity in circulating cells might have led to the neutropenia. Invernizzi et al. [4], Sahota et al. [5], and Kino et al. [6] showed that neutrophil counts dropped after several weeks of pregabalin use and recovered within one week after discontinuing pregabalin. In each of these reports, the presumption was that pregabalin-induced neutropenia was caused by direct damage to myeloid cells, and that the severity of damage was dose-dependent. As in previous reports of pregabalin-induced neutropenia, the pathology resolved within one week after cessation of mirogabalin in our case. As the elimination half-lives of pregabalin and mirogabalin are approximately six and three hours, respectively, we speculate that neutropenia caused by mirogabalin recovers faster than that caused by pregabalin after the respective drugs are discontinued. In drug-induced neutropenia resulting from direct damage to myeloid cells, the pathology usually develops 19-60 days after starting pregabalin treatment [7]. Neutropenia in this case occurred seven weeks after starting mirogabalin, which was relatively late for this adverse effect. We suggest that this might be because the dose of mirogabalin administered was low.

The incidence of all grades of neutropenia caused by immune-checkpoint inhibitors (ICIs) is reportedly 0.13%-0.94% [8-10]. Patients experiencing ICI-induced neutropenia require high-dose corticosteroid therapy for an average of 14 days in order to recover, and these individuals also suffer from other immune-related adverse events such as rash or colitis. Because our patient did not have any of these clinical symptoms, we feel it is unlikely that nivolumab (the ICI she was taking) was the cause of neutropenia.

Although amoxicillin-induced neutropenia has been reported, this drug is unlikely to have caused neutropenia in our case for the following reasons. First, the dose of amoxicillin was low (1,500 mg/day); second, the duration of administration was short (five days); and third, neutropenia occurred 12 days after the end of amoxicillin administration.

Both acetaminophen and mexiletine have been reported to cause neutropenia. However, since we did not observe neutropenia following restarting either of these drugs, we conclude that these agents are not the causative agents of neutropenia in our case.

## Conclusions

The neutropenia in the above case was most likely caused by mirogabalin. This drug is widely used for peripheral neuropathic pain resulting from pathologies such as diabetes, post-herpetic neuralgia and cancer. Although rare, mirogabalin-induced neutropenia is an adverse effect that healthcare providers should keep in mind when prescribing this drug.
